# Evaluation of the multifunctional dipeptidyl-peptidase IV and angiotensin converting enzyme inhibitory properties of a casein hydrolysate using cell-free and cell-based assays

**DOI:** 10.3389/fnut.2023.1198258

**Published:** 2023-05-22

**Authors:** Jianqiang Li, Carlotta Bollati, Gilda Aiello, Martina Bartolomei, Fabrizio Rivardo, Giovanna Boschin, Anna Arnoldi, Carmen Lammi

**Affiliations:** ^1^Department of Pharmaceutical Sciences, University of Milan, Milan, Italy; ^2^Department of Human Science and Quality of Life Promotion, Telematic University San Raffaele, Rome, Italy; ^3^A. Costantino & C. Spa, Favria, Italy

**Keywords:** ACE, anti-diabetic property, hypotensive property, casein hydrolysate, DPP-IV

## Abstract

The objective of the study was the evaluation of the potential pleiotropic effect of a commercial casein hydrolysate (CH). After an analysis of the composition, the BIOPEP-UWM database suggested that these peptides contained numerous sequences with potential inhibitory activities on angiotensin converting enzyme (ACE) and dipeptidyl-peptidase IV (DPP-IV). The anti-diabetic and anti-hypertensive effects of these peptides were thus assessed using either cell-free or cell-based assays. In the cell-free system, CH displayed inhibitory properties against DPP-IV (IC_50_ value equal to 0.38 ± 0.01 mg/mL) and ACE (IC_50_ value equal to 0.39 ± 0.01 mg/mL). Further, CH reduced the DPP-IV and ACE activities expressed by human intestinal Caco-2 cells by 61.10 ± 1.70% and 76.90 ± 4.47%, respectively, versus untreated cells, after 6 h of treatment at the concentration of 5 mg/mL. This first demonstration of the multifunctional behavior of this material suggests that it may become an anti-diabetic and/or anti-hypertensive ingredient to be included in the formulation of different functional food or nutraceutics.

## 1. Introduction

Type II diabetes is a chronic disease, which affects millions of people worldwide: the inadequate secretion of insulin by β-cells and/or insulin resistance in tissues are its main characteristics (1). Dipeptidyl-peptidase IV (DPP-IV) is known for its inactivation of two intestinal hormones, including glucagon-like peptide-1 (GLP-1) and glucose-dependent insulinotropic polypeptide (GIP), which possess potent insulin-secretory activity, and consequently lower prandial plasma glucose (1). Due to the short half-life (< 2 min) of the degradation of GLP-1 and GIP by DPP-IV, the inhibition of DPP-IV is an attractive therapeutic strategy to maintain the insulinotropic activity of GLP-1 and GIP, resulting in an improved homeostasis of glucose in type II diabetes (2). Interestingly, a number of studies indicate that most people with diabetes will eventually develop hypertension and other blood circulation complications, especially showing a close association between Type II diabetes and hypertension (3, 4). There are many factors associated with the development of hypertension, one of them is related to the conversion of angiotensin I, an active decapeptide, into angiotensin II, a potent vasoconstrictor octapeptide, by the angiotensin-converting enzyme (ACE) system. Meanwhile, the inactivation of bradykinin, which shows a significant vasodilator activity, is caused by ACE. Therefore, inhibition of ACE is considered an effective treatment for lowering blood pressure in hypertensive subjects (5).

Inhibitors of DPP-IV and/or ACE are regularly applied in therapy to lower morbidity and mortality of patients with type II diabetes and/or hypertension. Synthetic inhibitors of DPP-IV and/or ACE are the first-line option for the treatment of these events (6), however, inevitable and evitable side effects of them, such as coughing, skin rashes, taste disturbances, angioedema, hyperkalaemia (ACE inhibitors) (7, 8), and nasopharyngitis, headache, diarrhea, joint pain, urinary tract infection (DPP-IV inhibitors) (9, 10), highlight the importance of developing new natural, innovative side-effect free compounds to prevent and/or alleviate diabetes and hypertension. Over the past few decades, many evidences have underlined that protein hydrolysates obtained from both plant (soybean, pea, rice bran, lupin, wheat, and many others) and/or animal (milk, egg yolk, beef, pork, chicken, sardine, salmon, and many others) foods may be useful for the prevention of these pathologies and several food derived peptides with potent DPP-IV-inhibitory and/or ACE-inhibitory activities have been successfully identified and characterized (11–16). These peptides are encrypted within the protein sequences and are released by the hydrolysis (17). They may be obtained with digestive enzymes (i.e., pepsin and trypsin), mimicking the action of *in vivo* gastrointestinal digestion, or produced using plant (i.e., bromelin) or microbial (i.e., Alcalase® and Flavourzyme®) food grade enzymes, as well as during some food manufacture processes, such fermentation (18, 19).

It has been clearly demonstrated (20) that milk protein derived peptides possess different biological properties, including antimicrobial (21), antioxidant (22), anti-inflammatory (23), antithrombotic, hypocholesterolemic (24) and anti-hypertensive (22) activities useful for cardiovascular risk prevention. Moreover, they are active in preventing nervous system and bone diseases (25, 26). Most of these bioactive peptides are generated from the degradation of casein, which makes up around 80% of the protein in cow milk and it is composed by α-, β- and κ-caseins (27).

In light with these considerations, this study had the goal of assessing the pleiotropic health-promoting effects of a commercial casein hydrolysate (CH), obtained by an industrial process. Since casein is an excellent source of biologically active peptides, it was hypothesized that this industrial product may possess a dual biological behavior, including DPP-IV-inhibitory and/or ACE-inhibitory activity. The first objective of the study was the characterization of the chemical composition of CH for identifying sequences that may be responsible for its biological activity. The second aim was the evaluation of the potential anti-diabetic and hypotensive effects of CH by measuring its inhibitory activities toward both DPP-IV and ACE. This was achieved by using either cell-free tests or cell assays based on human intestinal Caco-2 cells. Finally, the same experimental procedures were employed to evaluate the activities of the fraction with a molecular weight lower than 3 kDa [CH (F3)].

## 2. Results

### 2.1. Peptidomic characterization of CH

**Figure 1:**
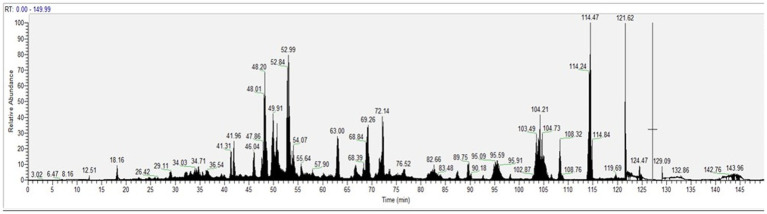
The total ion chromatogram (TIC) of CH was obtained injected 1 μg/μL.

The peptide composition of CH was analyzed by using HPLC-ESI-MS/MS. Figure 1 displays the total ion current (TIC) of the MS/MS of eluted peptides. The percentage of peptides with MW > 3 kDa was 37.95%, whereas the percentage of peptides with MW < 3 kDa was 62.05%. In addition, CH was ultra-filtered using a 3 kDa cutoff obtaining the low molecular peptide fraction, named CH (F3), which was investigated in parallel with total CH sample.

As shown in Table 1, 37 identified peptides belong to caseins: in particular, 18 belong to α-S1-casein, whereas 19 belong to β-casein. The lengths of those peptides range from 6 to 18 amino acids, whereas their isoelectric points (pI) and hydrophobicity range from 2.87 to 11.13 (pH) and from +4.41 to +23.87 (kcal×mol^−1^), respectively. Additionally, as reported by the BIOPEP-UWM database (,^1^ accessed on 16 Feb 2023), all peptides contain one or more motives including known sequences of either ACE or DPP-IV inhibitors.

**Table 1:** Identified peptides belonging to caseins, with inhibitory activity against both angiotensin converting enzyme (ACE) and dipeptidyl peptidase-IV (DPP-IV).

Protein name	Peptide sequence	Modifications	Theo. MH+ [Da]	Isoelectric point (pI)	Hydrophobicity (Kcal*mol^−1^)^a^	ACE inhibitor sequence^b^	DPP-IV inhibitor sequence^b^
α-S1-casein [OS=*Bos taurus*]	DIPNPIGSE		941,45745	2.87	+15.67	IP, IG, GS	IP, NP, PI, PN
	DIPNPIGSEN		1055,50038	2.89	+16.52	IP, IG, GS	IP, NP, PI, PN
	DIPNPIGSENSEK		1399,66996	3.73	+23.41	IP, IG, GS, EK	IP, NP, EK, PI, PN
	EDVPSER		831,38429	3.72	+20.75	VP, ER	VP, PS
	IPNPIGSE		826,43051	3.09	+12.03	IP, IG, GS	IP, NP, PI, PN
	IPNPIGSENSEK		1284,64302	4.08	+19.77	IP, IG, GS, EK	IP, NP, EK, PI, PN
	IPNPIGSENSEKTTMP	1xOxidation [M15]	1730,82654	4.08	+19.74	IP, IG, GS, EK	MP, IP, NP, EK, KT, PI, PN, TM, TT
	KEDVPSERY		1122,54258	4.33	+22.84	RY, VP, KE, KEDVPSE, ER	VP, KE, PS
	KEPMIGVN	1xOxidation [M4]	903,46043	6.53	+14.22	IG, GV, KE	EP, GV, KE, MI, PM, VN
	LEQILR		771,47231	6.84	+10.49	IL, LR	IL, QI
	SDIPNPIGSE		1028,48948	2.87	+16.13	IP, IG, GS	IP, NP, PI, PN
	SDIPNPIGSEN		1142,53241	2.89	+16.98	IP, IG, GS	IP, NP, PI, PN
	SDIPNPIGSENS		1229,56444	2.92	+17.44	IP, IG, GS	IP, NP, PI, PN
	SDIPNPIGSENSE		1358,60703	2.79	+21.07	IP, IG, GS	IP, NP, PI, PN
	SDIPNPIGSENSEK		1486,70199	3.73	+23.87	IP, IG, GS, EK	IP, NP, EK, PI, PN
	SDIPNPIGSENSEKTT		1688,79735	3.73	+24.37	IP, IG, GS, EK	IP, NP, EK, KT, PI, PN, TT
	SDIPNPIGSENSEKTTMP	1xOxidation [M17]	1932,88551	3.73	+23.84	IP, IG, GS, EK	MP, IP, NP, EK, KT, PI, PN, TM, TT
	VPNSAEER	1xPhospho [S4]	981,40372	4.08	+18.46	VP, ER	VP, AE, PN
β-casein [OS=*Bos taurus*]	EMPFPKYP	1xOxidation [M2]	1024,48083	6.67	+11.66	FP, EMPFPK, YP, KY, PFP	MP, FP, YP, KY, PF, PK, MPF
	EMPFPKYPVEP	1xOxidation [M2]	1349,64460	4.08	+14.97	FP, EMPFPK, YP, KY, VE, PFP	MP, FP, YP, EP, KY, PF, PK, PV, VE, MPF
	EPVLGPVRGPFP		1264,70483	6.72	+12.32	FP, LGP, GP, GPV, LG, VR, RG, LGPVRGPFP, VRGP, PFP	GP, FP, EP, VLGP, VR, PF, PV, RG, VL, GPV, GPF
	FPKYPVE		879,46108	6.61	+11.73	FP, YP, KY, VE	FP, YP, KY, PK, PV, VE
	FPKYPVEP		976,51384	6.61	+11.87	FP, YP, KY, VE	FP, YP, EP, KY, PK, PV, VE
	GPVRGPFP		826,45700	11.13	+10.26	FP, GP, GPV, VR, RG, VRGP, PFP	GP, FP, VR, PF, PV, RG, GPV, GPF
	GPVRGPFPII		1052,62513	11.13	+8.02	FP, GP, GPV, VR, GPVRGPFPII, RG, VRGP, PFP	GP, FP, VR, II, PF, PI, PV, RG, GPV, GPF
	GPVRGPFPIIV		1151,69354	11.03	+7.56	FP, GP, GPV, VRGPFPIIV, VR, GPFPIIV, RGPFPIIV, GPVRGPFPII, FPIIV, RG, VRGP, PFP	GP, FP, VR, II, PF, PI, PV, RG, GPV, GPF
	IPPLTQTPVVVP		1260,75620	5.23	+5.98	IPP, PL, IP, VP, TQ, PP, TP, PPL, IPPLTQTPV	PP, VP, VV, IP, TP, PL, PPL, IPPLTQTPV, LT, PV, QT, TQ
	IPPLTQTPVVVPP		1357,80896	5.23	+6.12	IPP, VPP, PL, IP, VP, TQ, PP, VVPP, TP, PPL, IPPLTQTPV	PP, VP, VV, IP, TP, PL, PPL, IPPLTQTPV, LT, PV, QT, TQ
	IPPLTQTPVVVPPF		1504,87738	5.46	+4.41	IPP, VPP, PL, IP, VP, TQ, PP, VVPP, TP, LTQTPVVVPPF, VVVPPF, PPL, IPPLTQTPV	PP, VP, VV, IP, TP, PL, PPL, IPPLTQTPV, LT, PF, PV, QT, TQ
	MPFPKYPVE	1xOxidation [M1]	1123,54924	6.60	+11.20	FP, YP, KY, VE, PFP	MP, FP, YP, KY, PF, PK, PV, VE, MPF
	MPFPKYPVEP		1204,60709	6.60	+11.34	FP, YP, KY, VE, PFP	MP, FP, YP, EP, KY, PF, PK, PV, VE, MPF
	MPFPKYPVEP	1xOxidation [M1]	1220,60201	6.60	+11.34	FP, YP, KY, VE, PFP	MP, FP, YP, EP, KY, PF, PK, PV, VE, MPF
	NIPPLTQTPVVVPP		1471,85189	5.09	+6.97	IPP, VPP, NIPPLTQTPV, PL, IP, VP, TQ, PP, VVPP, TP, PPL, IPPLTQTPV	PP, VP, VV, IP, TP, PL, PPL, IPPLTQTPV, LT, PV, QT, TQ
	PLTQTPVVVPP		1147,67214	5.25	+7.10	VPP, PL, VP, TQ, PP, VVPP, TP	PP, VP, VV, TP, PL, LT, PV, QT, TQ
	QEPVLGPVRGPFP		1392,76341	6.57	+13.09	FP, LGP, GP, GPV, LG, VR, RG, QEPVLGPVRGPFP, LGPVRGPFP, VRGP, PFP	GP, FP, EP, VLGP, VR, PF, PV, QE, RG, VL, GPV, GPF
	VLGPVRGPFP		1038,60948	11.11	+8.55	FP, LGP, GP, GPV, LG, VR, RG, LGPVRGPFP, VRGP, PFP	GP, FP, VLGP, VR, PF, PV, RG, VL, GPV, GPF
	YQEPVLGPVRGPFP		1555,82674	6.58	+12.38	FP, LGP, GP, GPV, LG, VR, RG, YQEPVL, QEPVLGPVRGPFP, LGPVRGPFP, VRGP, PFP	GP, FP, EP, VLGP, VR, PF, PV, QE, RG, VL, YQ, GPV, GPF

a According to PepDraw (http://pepdraw.com).

b According to the BIOPEP-UWM database; https://biochemia.uwm.edu.pl/biopep-uwm/ accessed on 16 Feb 2023.

### 2.2. Biochemical study of the DPP-IV and ACE inhibitory activities of CH

#### 2.2.1. The inhibition of *in vitro* DPP-IV activity by CH and CH (F3)

**Figure 2:**
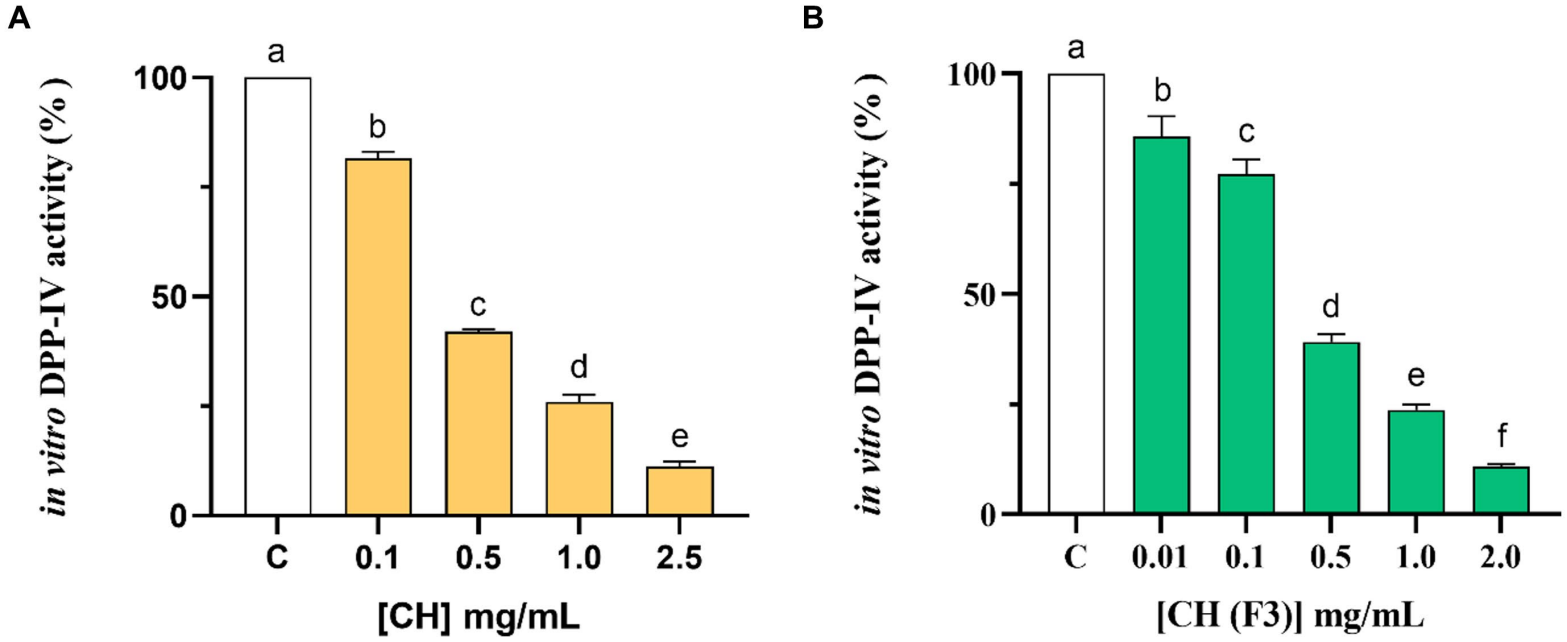
Examining the *in vitro* inhibitory properties of CH **(A)** and CH (F3) **(B)** against human recombinant dipeptidyl peptidase-IV (DPP-IV), obtained using cell-free assay. The bars show the mean ± s.d. of three independent experiments carried out in duplicate. Different lowercase letters indicate a significant difference (*p* < 0.05) between different concentrations. C (H_2_O): control cells.

The assessment of CH ability to regulate the DPP-IV activity was carried out using the purified recombinant DPP-IV enzyme. Results clearly demonstrated that CH decreased the DPP-IV activity *in vitro* with a dose–response trend in the concentration range of 0.1, 0.5, 1.0, and 2.5 mg/mL, displaying an IC_50_ value of 0.38 ± 0.01 mg/mL (Figure 2A). To further confirm this inhibitory activity, the experiments were carried out on the low molecular weight fraction (<3 kDa), named CH (F3), in the same experimental conditions (Figure 2B). This low molecular weight fraction dropped the DPP-IV activity *in vitro* too, showing a dose–response trend and an IC_50_ value of 0.31 ± 0.05 mg/mL (Figure 2B). A comparison of the IC_50_ value of the total CH hydrolysate with that of CH (F3) suggests that short-sized peptides are mainly responsible for the DPP-IV inhibitory activity (Table 2).

**Table 2:** IC_50_ values obtained testing CH and its fractions (molecular weight: < 3 kDa) against *in vitro* dipeptidyl peptidase-IV (DPP-IV) and angiotensin converting enzyme (ACE).

Hydrolysate	IC_50_ (mg/mL) DPP-IV	IC_50_ (mg/mL) ACE
CH	0.38 ± 0.01	0.39 ± 0.01
CH < 3 kDa (F3)	0.31 ± 0.05	0.24 ± 0.01

#### 2.2.2. Effect of CH and CH (F3) on *in vitro* ACE activity

**Figure 3:**
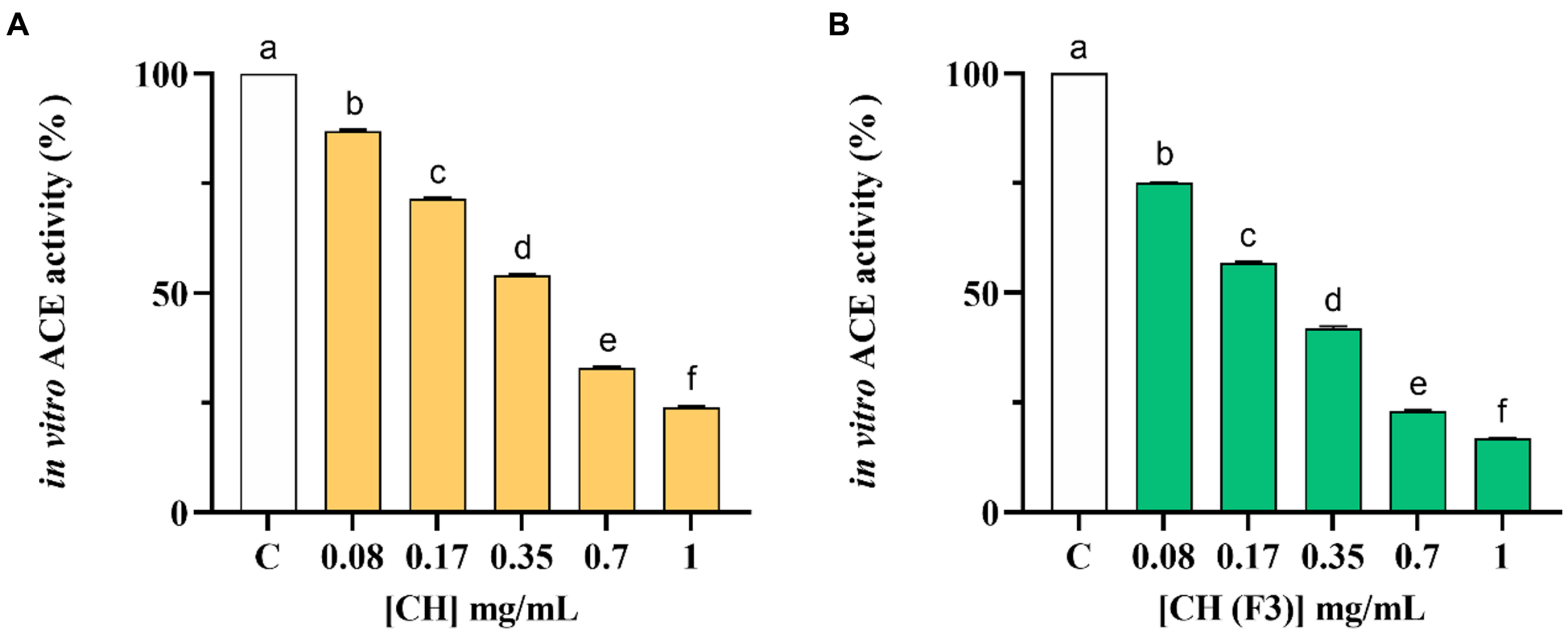
The inhibitory effects of CH **(A)** and CH (F3) **(B)** on angiotensin converting enzyme (ACE) were evaluated *in vitro*, using cell-free assay. The bars representing the mean ± s.d. of three independent experiments performed in duplicate. Different lowercase letters indicate a significant difference (*p* < 0.05) between different concentrations. C (H_2_O): control cells.

The inhibitory effects of CH on the *in vitro* ACE activity were tested at 0.08–1.0 mg/mL concentration range. As shown in Figure 3A, CH efficiently inhibited the *in vitro* ACE activity with a dose–response trend and a calculated IC_50_ value of 0.39 ± 0.01 mg/mL, whereas CH (F3) displayed an IC_50_ value of 0.24 ± 0.01 mg/mL (Figure 3B). Interestingly, the inhibitory activities of CH (F3) were slightly higher than that of CH at the same concentrations, indicating that short peptides are probably responsible for the *in vitro* ACE inhibitory activity.

Finally, Table 2 summarizes a comparison of the *in vitro* IC_50_ values of CH and CH (F3) against DPP-IV and ACE.

### 2.3. The examination of DPP-IV and ACE inhibition by CH and CH (F3) at cellular level

#### 2.3.1. CH and CH (F3) inhibit the DPP-IV activity expressed by Caco-2 cells

**Figure 4:**
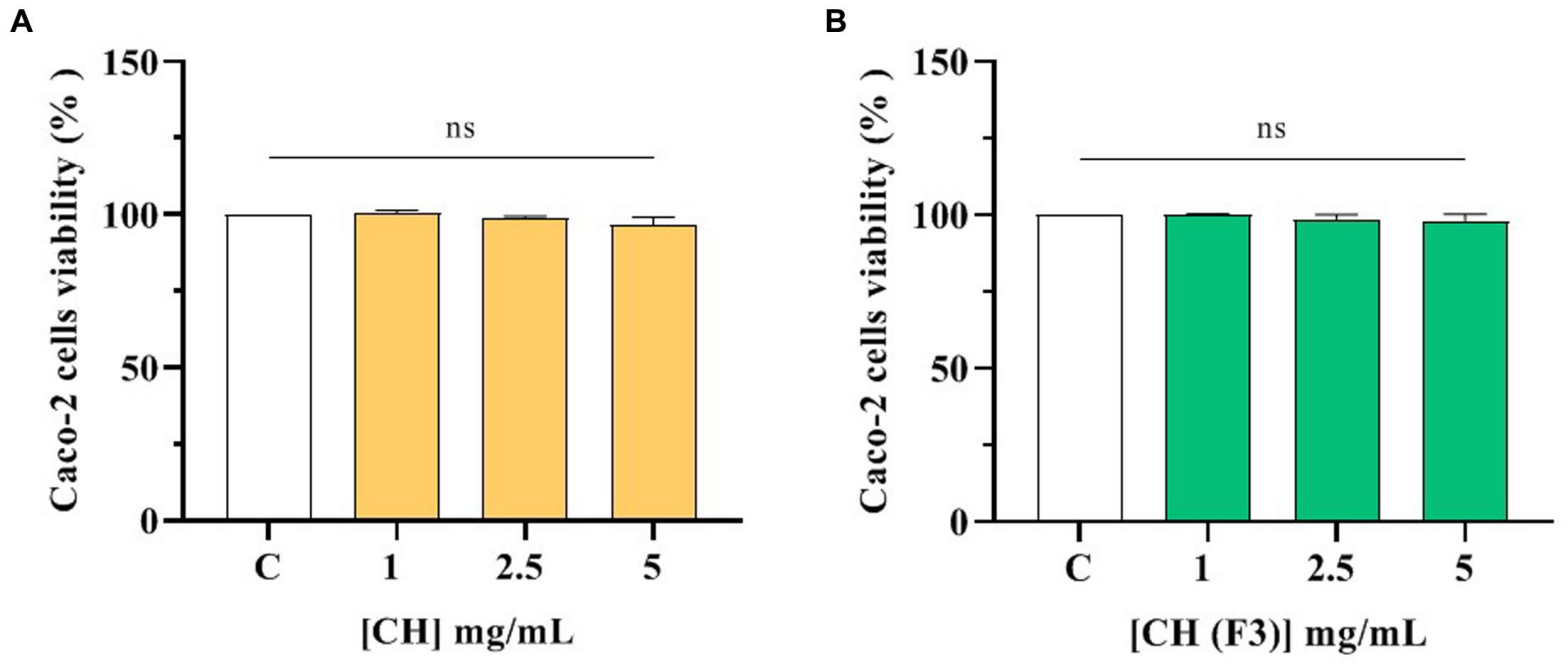
Effect of CH **(A)** and CH (F3) **(B)** on the Caco-2 cells viability. Data represents the mean ± s.d. of four independent experiments carried out in duplicate. ns: no significance, C: control cells (H_2_O).

No effects of CH and CH (F3) on human intestinal Caco-2 cells viability was observed by performing MTT after 48-h treatment within the concentration range of 1.0–5.0 mg/mL compared with control cells (C) (Figure 4), suggesting that both samples are safe in the range of doses tested.

**Figure 5:**
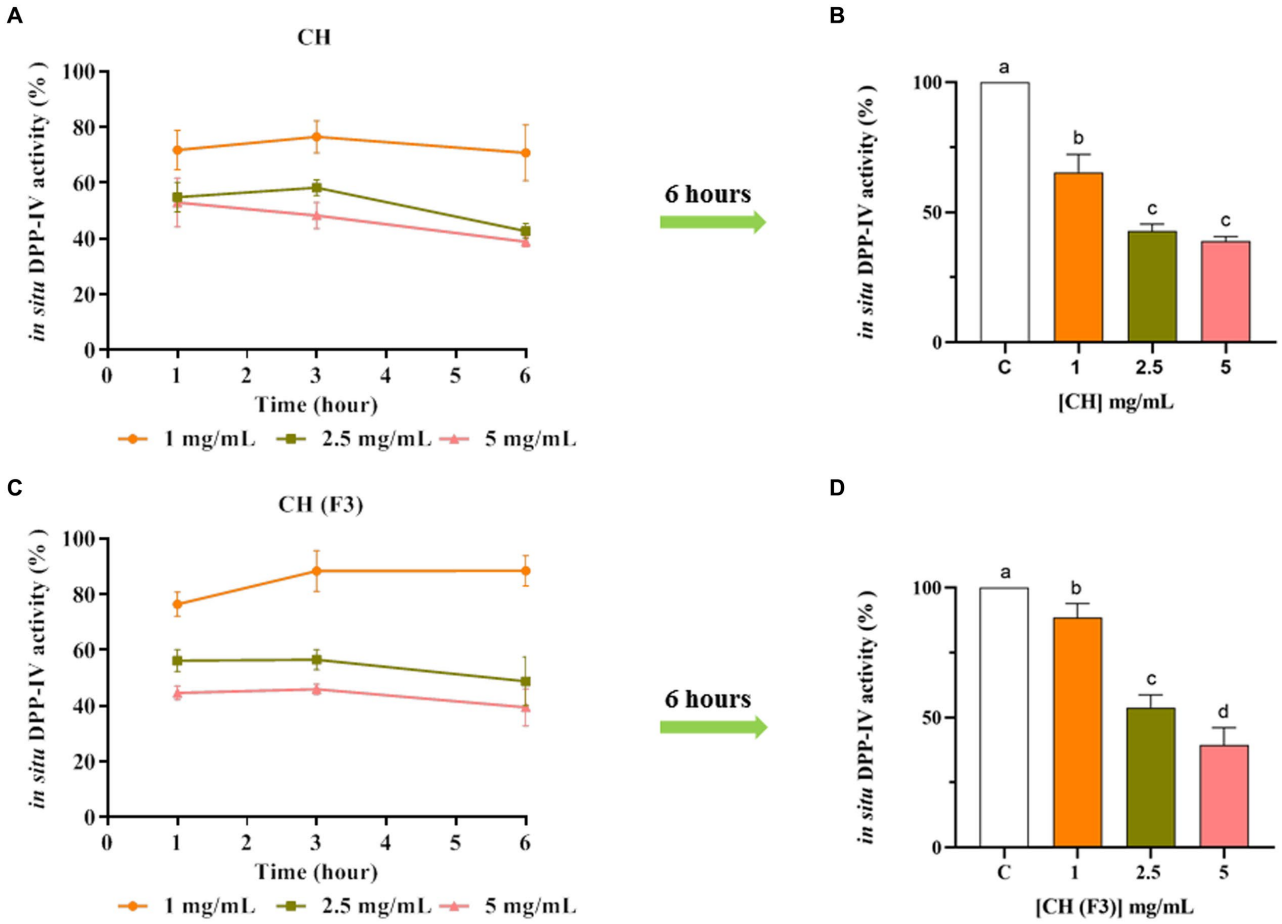
The effect of CH **(A)** and CH (F3) **(B)** on cellular dipeptidyl peptidase-IV (DPP-IV) activity was determined, and the mean ± s.d. of four independent experiments performed in triplicate are displayed as data points. Different lowercase letters indicate a significant difference (*p* < 0.05) between different concentrations. ns: no significance; C (H_2_O): control cells.

The CH inhibitory effect of DPP-IV expressed on Caco-2 cellular membranes was then investigated. In detail, the inhibitory effect of CH and CH (F3) on cellular DPP-IV activity was measured in a kinetic mode after 1, 3, and 6 h of incubation with CH or CH (F3) in the range of concentrations 1.0–5.0 mg/mL. As shown in Figure 5A, CH dropped the cellular DPP-IV activity by 28.20 ± 7.11%, 45.11 ± 5.38%, and 47.04 ± 8.73% at 1.0, 2.5 and 5 mg/mL, respectively (after 1 h), and by 23.46 ± 5.79%, 41.73 ± 2.88% and 51.68 ± 4.71% at the same concentrations (after 3 h). However, the maximal inhibition of cellular DPP-IV activity was observed after 6 h (Figures 5A,B), when CH inhibited the cellular enzyme activity by 29.23 ± 9.98%, 57.26 ± 2.69%, and 61.10 ± 1.70% at 1.0, 2.5 and 5 mg/mL, respectively, compared with untreated cells, displaying a dose–response trend and confirming the *in vitro* results.

In parallel, the evaluation of CH (F3) inhibitory activity of cellular DPP-IV was carried. Notably, CH (F3) reduced the cellular DPP-IV activity by 23.55 ± 4.31%, 43.82 ± 3.98%, and 55.35 ± 2.55% at 1.0, 2.5 and 5 mg/mL (1 h), respectively, and by 11.63 ± 7.27%, 43.50 ± 3.59%, and 54.07 ± 1.87% at 3 h (Figure 5C). In line with CH, the best inhibition of cellular DPP-IV activity by CH (F3) was detected at 6 h (Figures 5C,D), when it dropped the cellular activity by 11.54 ± 5.39%, 51.22 ± 8.74%, and 60.53 ± 6.68% at 1.0, 2.5, and 5 mg/mL, respectively, versus untreated cells.

#### 2.3.2. CH and CH (F3) inhibit ACE activity expressed by Caco-2 cells

**Figure 6:**
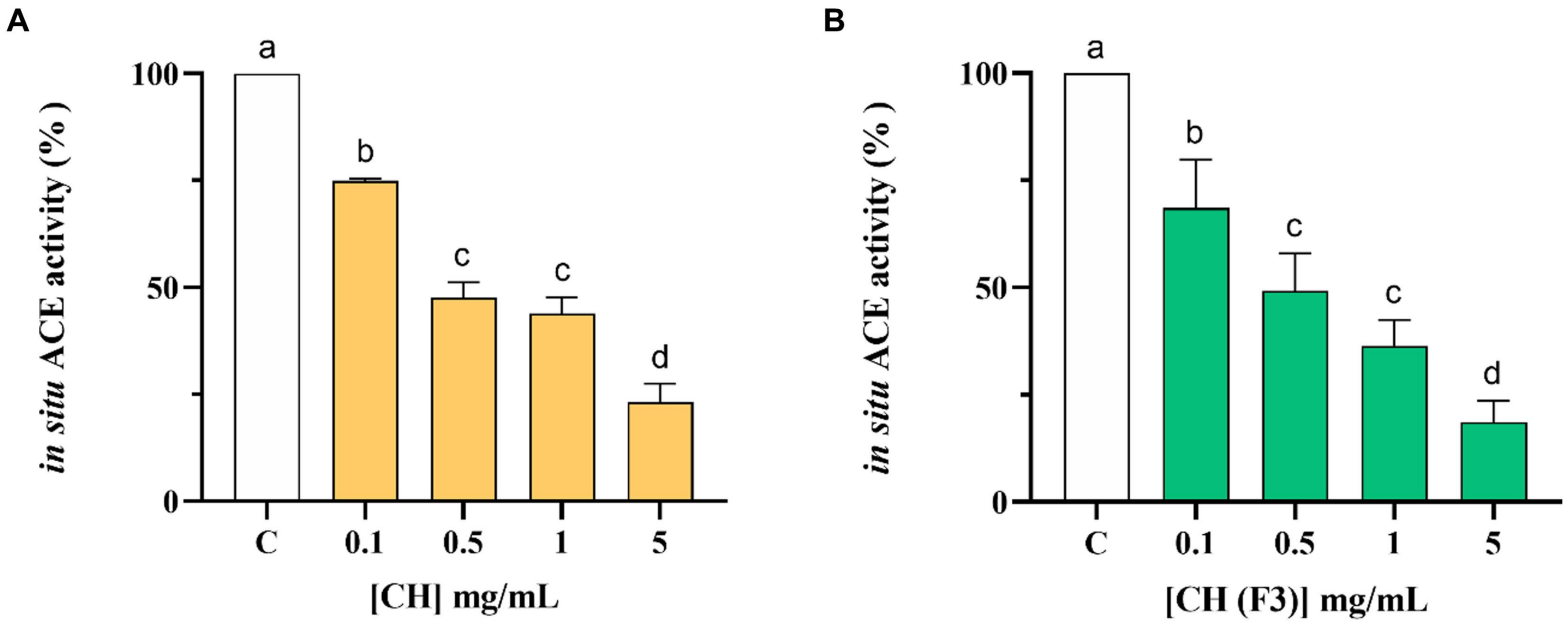
Evaluation of the inhibitory effect of CH **(A)** and CH (F3) **(B)** on angiotensin converting enzyme (ACE) expressed on Caco-2 cells membranes. Bars represent the mean ± s.d. of three independent experiments in triplicate. Different lowercase letters indicate a significant difference (*p* < 0.05) between different concentrations. ns: no significance; C (H_2_O): control cells.

The CH inhibitory effect on the ACE expressed on the membranes of Caco-2 cells was evaluated treating cells with CH at 1.0–5.0 mg/mL for 6 h. Results indicated that CH inhibited the cellular ACE activity with a dose–response manner, reducing the enzyme activity by 25.04 ± 0.49%, 52.36 ± 3.53%, 56.10 ± 3.78%, and 76.90 ± 4.47%, respectively, at 0.1, 0.5, 1.0 and 5.0 mg/mL (Figure 6A). Meanwhile, CH (F3) is able to decrease the cellular ACE activity by 31.42 ± 11.19%, 50.75 ± 8.75%, 63.63 ± 6.08%, and 81.39 ± 5.05% at 0.1, 0.5, 1.0, and 5.0 mg/mL after 6 h, respectively (Figure 6B).

## 3. Discussion

Undoubtedly, casein is among the most studied sources of bioactive peptides with health promoting activities. Indeed, several literature evidences report that casein acts as a precursor of a diversity of bioactive peptides with anti-hypertensive, immunological, antioxidant, antithrombotic and anti-diabetic effects (28–30). In this context, the ACE-inhibitory activity was the most investigated, and numerous ACE-inhibitory peptides have been identified, for instance, VPP (31), IPP (32), NMAINPSKENLCSTFCK (33), RYPSYG and DERF (34). In addition, also DPP-IV-inhibitory peptides have also been identified from casein and characterized, such as LPQNIPPL (35) and VPYPQ (36).

In this panorama, the main limitation of most previous studies on casein hydrolysate consists on the fact that the characterization of DPP-IV or ACE inhibitory activities has been carried out independently without taking into account that the same hydrolysate may be endowed with a multifunctional behavior exerting, therefore, both activities. Moreover, great part of published papers are based exclusively on cell-free assays (i.e., pure enzyme inhibition), whereas a main peculiarity of the present study is that the *in vitro* tests were implemented and substantiated by cellular assays that permitted either to get a deeper insight in the mechanism of action or to contextually consider other relevant issues, such as metabolism (this is particularly true while employing Caco-2 cells).

Overall, our findings suggested that CH is able to modulate the DPP-IV and ACE activities both in cell-free and cell-based conditions. More in details, CH displayed potent inhibitory properties against DPP-IV (IC_50_ value of 0.38 ± 0.01 mg/mL) and ACE (IC_50_ value of 0.39 ± 0.01 mg/mL) in cell-free condition, respectively. Furthermore, a comparison of the IC_50_ values of complete hydrolysate with those of the low molecular weight fraction (<3 kDa) (Table 2), showed that medium and short-sized peptides, which account for about two thirds of the hydrolysate (62.05%), are mainly responsible of the observed biological activities.

Literature suggests that a camel total casein hydrolysate is less efficient than CH in reducing the ACE activity being its effect not observed up to 0.6 mg/mL. Whereas, the <3 kDa fraction of the same material displays an IC_50_ equal to 91 ± 20 μg/mL (37). In addition, the IC_50_ values of a thermolysin casein hydrolysate was 45–83 μg/mL (38). As regards the DPP-IV inhibitory activity, previous evidences suggest that casein hydrolysates showed IC_50_ values in the range 0.88–1.1 mg/mL (39), suggesting that CH is slightly more active. In addition, as reported by Zhang et al., using different proteases, casein proteins released peptides that determined the 50% of DPP-IV inhibition at 1.25 μg/mL, clearly suggesting that CH is more active (40). Notably, recent evidences suggest that both soybean hydrolysate and pea hydrolysate inhibit DPP-IV and ACE activity with a dose–response trend and IC_50_ values equal to 1.15 ± 0.004 and 1.33 ± 0.004 mg/mL, and 0.33 ± 0.01 and 0.61 ± 0.05 mg/mL, respectively (11).

In principle, the bioactivity of a food derived hydrolysate depends strictly on its total composition, including inactive and active species and possible synergistic or antagonist effects (41, 42). In light with this observation, Table 1 reveals that all the major identified peptides derived from CH included at least one motif with established DPP-IV and ACE inhibitory activity, which clarifies the reason why this peptide mixture is effective against both targets. Being the composition of casein hydrolysate naturally heterogeneous, it is feasible to consider that some peptides may be active against one of the two target, but we cannot exclude that some specific peptides may be contemporary endowed by the dual ability to target both ACE and DPP-IV. In addition, some peptides with post-translational modifications were also identified. Notably, it was observed that peptide VPNSAEER displays a phosphorylation on serine residue in position 4, whereas 6 different peptides (IPNPIGSENSEKTTMP, KEPMIGVN, SDIPNPIGSENSEKTTMP, EMPFPKYP, EMPFPKYPVEP, and MPFPKYPVEP) contain within their sequences oxidized methionine residue.

These results are in line with a previous work which focused on two commercially available soybean (SH) and pea (PH) protein hydrolysates (11). In particular, as regards the ACE inhibitory activity, CH displayed comparable IC_50_ values with those obtained of both SH and PH, whereas as regards DPP-IV activity, CH hydrolysates is about 3.5-fold more potent than both SH and PH (11).

DPP-IV and ACE are vital membrane peptidases expressed physiologically by many tissues, such as the intestine (43, 44). As a matter of fact, human intestinal Caco-2 cells offer a dependable model that has already been established and confirmed for the investigation of peptides possessing DPP-IV or ACE inhibitory characteristics (45–47). Our findings indicated that CH maintains its ability to modulate the activity of both DPP-IV and ACE on Caco-2 cells, even though both hydrolysates are active in the range of concentration between 0.1 and 5 mg/mL as a function of the time (Figure 4A). In particular, CH inhibits both enzymes activity by 61.10 ± 1.70% and 76.90 ± 4.47% at the concentration of 5 mg/mL after 6 h of treatment (Figures 4,5), versus untreated cells, respectively, indicating that CH is less active in cell-based than in cell-free conditions. Similar results have been previously obtained on SH, PH, and peptic and tryptic hydrolysates of *Arthrospira platensis* (*Spirulina*) and *Chlorella pyrenoidosa* proteins, respectively (11, 45, 46, 48, 49). Also in these cases, the tested hydrolysates showed greater activity in cell-free assays than in cell-based assays. More in details, PH and SH inhibited the cellular DPP-IV activity by 53.6 ± 2.3% and 48.69 ± 7.29%, respectively, and the cellular ACE activity by 82.3 ± 4.2% and 74.6 ± 4.9%, respectively, at 5 mg/mL after 3 h (11). In addition, *C. pyrenoidosa* hydrolysates obtained using pepsin (CP) and trypsin (CT) inhibited the cellular ACE activity by 61.5 ± 7.7% and 69.9 ± 0.8%, respectively, at 5 mg/mL. When tested at the same concentration, they reduced the cellular DPP-IV activity by 38.4 and 42.5%, respectively (45).

This discrepancy in efficacy may be explained by the metabolic capabilities of Caco-2 cells, which express active proteases and peptidases that can actively metabolize peptides and impact their bioactivity (45). Thus, the intestine performs a dynamic function, not only in the process of vital nutrient absorption but also in actively modulating the physicochemical and biological profiles of food protein hydrolysates. Moreover, the peptides composition of CH here investigated results in line with a recent research dealing with the identification of novel casein-derived bioactive peptides and their potential (ACE)-inhibitory, antioxidant, and DPP-IV inhibitory activity when casein were fermented with *Lactobacillus helveticus* (50). Some of the identified peptides, i.e., DIPNPIGSE, EDVPSER, FPKYPVE are common to those generated by the fermentation.

## 4. Conclusion

For the first time, we have characterized the multifunctional behavior of a commercial casein hydrolysate, demonstrating its dual inhibitory properties on DPP-IV and ACE, using both cell-free and cell-based assays. Indeed, the active intestinal peptidases, which are expressed on cellular membrane, modulate peptide profile without impair their pleotropic activity. Hence, our findings constitute a significant foundation for future investigation of CH by *in vivo* and clinical studies for confirming its multifunctional and pleotropic behavior. With its ability to confer both hypotensive and antidiabetic benefits, CH may be a highly promising ingredient for use in the development of functional foods and dietary supplements aimed at preventing cardiovascular disease and metabolic syndrome.

## 5. Materials and methods

### 5.1. Reagents

Dulbecco’s modified Eagle’s medium (DMEM), L-glutamine, fetal bovine serum (FBS), phosphate buffered saline (PBS), penicillin/streptomycin and 96-well plates were purchased from Euroclone (Milan, Italy). Acetonitrile (ACN), formic acid, 3-(4,5-dimethylthiazol-2-yl)-2,5-diphenyltetrazolium bromide (MTT), sitagliptin, Gly-Pro-amido-4-methylcoumarin hydrobromide (Gly-Pro-AMC) and ACE from porcine kidney were from Sigma-Aldrich (St. Louis, MO, United States). ACE1 Activity Assay Kits come from Biovision (Milpitas Blvd., Milpitias, CA, United States). The DPP-IV inhibitor screening assay kit was bought from Cayman Chemical (Michigan, United States). Caco-2 cells were obtained from INSERM (Paris, France). Casein hydrolysates (CH), which is spray dried samples from the production process, was supplied by A. Costantino S.R.L. (Italy). (Casein hydrolysate sport/health batch 218F0004, 100 g). Casein hydrolysates (CH), which is spray dried samples from the production process, was supplied by A. Costantino S.R.L. (Italy). (Casein hydrolysate sport/health batch 218F0004, 100 g).

### 5.2. Ultrafiltration of CH

Prior to evaluating biological activity, CH was ultra-filtered using a 3 kDa cutoff on a Millipore ultrafiltration system (Millipore, Bedford, MA, United States). The peptide solution obtained was lyophilized (lyophilizer LIO5P, Cinquepascal S.r.l., Milan, Italy) for further analyzes after the storage temperature–80°C.

### 5.3. Mass spectrometry analysis

Samples were dissolved with 50 μL of a solution made of 1% acetonitrile (ACN) and 0.1% formic acid in distilled H_2_O. All samples were analyzed at UNITECH OMICs (University of Milan, Italy) using: Dionex Ultimate 3000 nano-LC system (Sunnyvale CA, United States) connected to Orbitrap Fusion™ Tribrid™ Mass Spectrometer (Thermo Scientific, Bremen, Germany) equipped with a nanoelectrospray ion source. Peptide mixtures were pre-concentrated onto an Acclaim PepMap 100–100 μm × 2 cm C18 (Thermo Scientific) and separated on EASY-Spray column ES900, 25 cm x 75 μm ID packed with Thermo Scientific Acclaim PepMap RSLC C18, 3 μm, 100 Å using mobile phase A (0.1% formic acid in distilled H_2_O) and mobile phase B (0.1% formic acid in acetonitrile 20/80, v/v) at a flow rate of 0.300 μL/min. The following gradient profile was used: 0 min, 4% B; 3 min, 4% B; 103 min, 28% B; 113 min, 95% B; 120 min, 4% B; 126 min, 95% B; 135 min, 4%B; 138 min, 95% B; 144 min, 4% B. The temperature was set to 35°C and the sample were injected in triplicates. MS spectra were collected over an *m/z* range of 375–1500 Da at 120,000 resolutions (*m/z* 200), operating in the data-dependent mode, cycle time of 3 s between masters scans. HCD was performed with collision energy set at 35 eV. Polarity: positive. MS data were analyzed by Proteome Discoverer 2.5 by using *Bos taurus* (sp_incl_isoforms TaxID = 9913_and_subtaxonomies) (v2022-12-14) without any specific enzymatic cut. Oxidation of methionine and acetylation at the protein N-terminus were specified as variable modifications.

### 5.4. Characterization of the inhibitory effects of CH on DPP-IV and ACE through biochemical assays

#### 5.4.1. *In vitro* assessment of DPP-IV-inhibitory activity

The *in vitro* assessment of DPP-IV-inhibitory activity was performed following the manufacturer instructions (DPP-IV inhibitor screening assay kit–Cayman Chemical) and previously reported methods (47, 48). The experiments were carried out in triplicate in a half–area 96 well solid plate (white). Each reaction (50 μL) was prepared adding the reagents in the following order in a microcentrifuge tube: 1 X assay buffer [20 mM Tris–HCl, pH 8.0, containing 100 mM NaCl, and 1 mM EDTA] (30 μL), CH and CH (F3) at final concentration range of 0.01–2.0 mg/mL (10 μL) or vehicle (10 μL of distilled H_2_O) and finally the DPP-IV enzyme (10 μL). Subsequently, the samples were mixed and 50 μL of each reaction were transferred in each well of the plate. The reactions were started by adding 50 μL of substrate solution to each well and incubated at 37°C for 30 min. Fluorescence signals were measured using the Synergy H1 fluorescent plate reader from Biotek (excitation and emission wavelengths 360 and 465 nm, respectively).

#### 5.4.2. *In vitro* assessment of ACE-inhibitory activity

The ACE-inhibitory activity of the CH was evaluated following previously reported methods (51, 52) and tested by measuring with HPLC the formation of hippuric acid (HA) from hippuryl-histidyl-leucine (HHL), used as mimic substrate for ACE. Tests were performed in 100 mM Tris-HCOOH, 300 mM NaCl pH 8.3 buffer, and using ACE from porcine kidney (Sigma-Aldrich, Milan, Italy). CH hydrolysate was tested at 0.08, 0.17, 0.35, 0.7 and 1.0 mg/mL.

### 5.5. Assessment of the inhibitory effects of CH on DPP-IV and ACE activities using cellular assays

#### 5.5.1. Culture of Caco-2 cells

Caco-2 cells were routinely sub-cultured at 50% density and maintained at 37°C in 5% CO_2_ atmosphere in Dulbecco’s modified Eagle’s medium (DMEM) containing 25 mM of glucose, 3.7 g/L of NaHCO_3_, 4 mM of stable L-glutamine, 1% nonessential amino acids, 100 U/L of penicillin, and 100 μg/L of streptomycin (complete medium), supplemented with 10% heat-inactivated fetal bovine serum (FBS), as reported elsewhere (53). The cells were used for no more than 20 passages.

#### 5.5.2. 3-(4,5-dimethylthiazol-2-yl)-2,5-diphenyltetrazolium bromide (MTT) assay

A total of 3 × 10^4^ Caco-2 cells/well were seeded in 96-well plates and, the day after, they were treated with 1.0, 2.5, and 5 mg/mL of CH or CH (F3) or vehicle (distilled H_2_O) in complete growth media for 48 h at 37°C under 5% CO_2_ atmosphere. Subsequently, the treatment solvent was aspirated and 100 μL/well of 3-(4,5-dimethylthiazol-2-yl)-2,5-diphenyltetrazolium bromide (MTT) filtered solution added. After 2 h of incubation at 37°C under 5% CO_2_ atmosphere, 0.5 mg/mL solution was aspirated and 100 μL/well of the lysis buffer (8 mM HCl + 0.5% NP-40 in DMSO) added. After 10 min of slow shaking, the absorbance at 575 nm was read on the Synergy H1 fluorescence plate reader (Biotek, Bad Friedrichshall, Germany) as reported in previously work (11).

#### 5.5.3. Assessment of the inhibitory activity of CH on cellular DPP-IV activity

Caco-2 cells were seeded on black 96-well plates with clear bottoms at a density of 3 × 10^4^ cells/well. After 1 day, cells were treated with 1.0, 2.5 and 5.0 mg/mL of CH or CH (F3) (100 μL/well) for 1, 3 and 6 h; at the end of the incubation, cells were washed once with 100 μL of PBS, then, 50 μL of Gly-Pro-AMC substrate at concentrations of 25 μM in PBS were added and the fluorescence signals (excitation/emission wavelengths 350/450 nm) in each well were measured using a Synergy H1 (BioTek Instruments, Winooski, VT, USA) every 1 min for 10 min.

#### 5.5.4. Assessment of the inhibitory activity of CH on cellular ACE1 activity

The inhibitory activity of CH on cellular ACE1 activity was evaluated following a previously reported method (54). Caco-2 cells were seeded on 96-well plates at a density of 3 × 10^4^ cells/well for 24 h. The following day, cells were treated with 100 μL/well of CH or CH (F3) (at the final concentrations of 0.1–5.0 mg/mL) or vehicle in growth medium for 6 h at 37°C, then cells were scraped in 30 μL of ice-cold ACE1 lysis buffer and transferred in an ice-cold eppendorf tube. After centrifugation at 13,300 *g* for 15 min at 4°C, the supernatant was recovered and transferred into a new ice-cold tube. Total proteins were quantified by Bradford method, and 2 μg of total proteins (the equivalent of 2 μL) were added to 18 μL of ACE1 lysis buffer in each well in a black 96-well plates with clear bottoms. For the background control, 20 μL of ACE1 lysis buffer were added to 20 μL of ACE1 assay buffer. Subsequently, 20 μL of 4% of ACE1 substrate (prepared in assay buffer) was added in each well except the background one and the fluorescence (Ex/Em 330/430 nm) was measured in a kinetic mode for 10 min at 37°C.

### 5.6. Statistical analysis

The statistical analysis was conducted through one-way ANOVA (GraphPad Prism 9.1, GraphPad Software, La Jolla, CA, United States), and Tukey’s multiple comparison test was used for post-hoc analysis. The assays were performed independently at least four times, and each experiment was conducted in triplicate. The data were expressed as mean ± standard deviation (s.d.), and statistical significance was set at *p* < 0.05.

## Data availability statement

The original contributions presented in the study are included in the article/Supplementary material, further inquiries can be directed to the corresponding author.

## Author contributions

CL, JL, CB, GA, MB, GB, and FR: experiments. CL: discussion of the results. CL and JL: manuscript writing (original draft). CL and AA: manuscript editing. All authors have read and agreed to the published version of the manuscript.

## Funding

This research and the APC were funded by A. Costantino & C. Spa.

## Conflict of interest

FR was employed by A. Costantino & C. Spa which had the following involvement in the study: provided the calculated molecular weight distribution of peptide samples.

The remaining authors declare that the research was conducted in the absence of any commercial or financial relationships that could be construed as a potential conflict of interest.

## Publisher’s note

All claims expressed in this article are solely those of the authors and do not necessarily represent those of their affiliated organizations, or those of the publisher, the editors and the reviewers. Any product that may be evaluated in this article, or claim that may be made by its manufacturer, is not guaranteed or endorsed by the publisher.

## Footnotes

^1^ https://bio-chemia.uwm.edu.pl/biopep-uwm/
